# Exo70 Protects Against Memory and Synaptic Impairments Following Mild Traumatic Brain Injury

**DOI:** 10.3390/antiox14060640

**Published:** 2025-05-26

**Authors:** Matías Lira, Jorge Abarca, Rodrigo G. Mira, Pedro Zamorano, Waldo Cerpa

**Affiliations:** 1 Laboratorio de Función y Patología Neuronal, Facultad de Ciencias Biológicas, Pontificia Universidad Católica de Chile, Santiago 8331150, Chile; mslira1@uc.cl (M.L.); jabarca@bio.puc.cl (J.A.); rnmira@uc.cl (R.G.M.); 2 Centro de Excelencia en Biomedicina de Magallanes (CEBIMA), Universidad de Magallanes, Punta Arenas 6210005, Chile; 3 Departamento Biomédico, Universidad de Antofagasta, Antofagasta 1271155, Chile; pedro.zamorano@uamail.cl; 4 Instituto Antofagasta, Universidad de Antofagasta, Antofagasta 1271155, Chile

**Keywords:** traumatic brain injury, exocyst, Exo70, NMDAR, LTP, memory

## Abstract

Mild traumatic brain injury (mTBI), a leading cause of disability in young adults, often results from external forces that damage the brain. Cellularly, mTBI induces oxidative stress, characterized by excessive reactive oxygen species (ROS) and diminished antioxidant capacity. This redox imbalance disrupts hippocampal glutamatergic transmission and synaptic plasticity, where NMDA receptors (NMDARs) are crucial. The exocyst, a vesicle tethering complex, is implicated in glutamate receptor trafficking. We previously showed that Exo70, a key exocyst subunit, redistributes within synapses and increases its interaction with the NMDAR subunit GluN2B following mTBI, suggesting a role in GluN2B distribution from synaptic to extrasynaptic sites. This study investigated whether Exo70 could mitigate mTBI pathology by modulating NMDAR trafficking under elevated oxidative stress. Using a modified Maryland mTBI mouse model, we overexpressed Exo70 in CA1 pyramidal neurons via lentiviral transduction. Exo70 overexpression prevented mTBI-induced cognitive impairment, assessed by the Morris water maze. Moreover, these mice exhibited basal and NMDAR-dependent hippocampal synaptic transmission comparable to sham animals, preventing mTBI-induced deterioration. Preserved long-term potentiation, abundant synaptic GluN2B-containing NMDARs, and downstream signaling indicated that Exo70 overexpression prevented mTBI-related alterations. Our findings highlight Exo70’s crucial role in NMDAR trafficking, potentially counteracting oxidative stress effects. The exocyst complex may be a critical component of the machinery regulating NMDAR distribution in health and disease, particularly in pathologies featuring oxidative stress and NMDAR dysfunction, like mTBI.

## 1. Introduction

Traumatic brain injury (TBI) is an alteration in brain function and physiology resulting from external forces that cause brain movement within the skull [1]. It is a critical health problem worldwide that leads to high medical costs [2] and is recognized as a significant cause of permanent disability and death among young adults [3], with approximately sixty-nine million individuals experiencing TBI worldwide [4]. The most frequent form is mild traumatic brain injury (mTBI), representing 80–90% of all TBI cases [5,6,7].

At the cellular level, mTBI triggers a cascade of events, primarily characterized by oxidative stress. This is characterized by an imbalance between the production of reactive oxygen species (ROS), such as superoxide radicals, hydrogen peroxide, and hydroxyl radicals, and the brain’s ability to detoxify these reactive intermediates or repair the resulting damage through its antioxidant defense systems [8]. Major antioxidant enzymes involved include superoxide dismutase (SOD), catalase (CAT), and glutathione peroxidase (GPx) [9]. Oxidative stress significantly contributes to secondary injury mechanisms in mTBI, leading to lipid peroxidation, protein oxidation, and DNA damage, ultimately resulting in neuronal dysfunction and cell death [10,11].

Despite being the most common, mTBI shares many of these cellular mechanisms, including oxidative stress, with moderate and severe TBI, exhibiting a broad spectrum of cellular responses that depend on the severity of the insult and pathophysiology [7]. These cellular alterations include axonal disruption, excitotoxicity, neuroinflammation, neuronal malfunction, and cell death, among others [7,12].

N-methyl-D-aspartate receptor (NMDAR) is a critical ionotropic glutamate receptor in synaptic plasticity and cognition [13]. It has been extensively studied in several traumatic brain injury (TBI) models using proteomic, electrophysiological, and behavioral approaches [7,12,14]. Basal synaptic transmission and plasticity decrease upon TBI induction [15,16], accompanied by a reduction in glutamate binding to receptors [17] and, ultimately, the internalization of these receptors. Significantly, oxidative stress can directly modify NMDAR function, altering its sensitivity to glutamate and contributing to excitotoxicity [18,19]. In addition, the NMDAR subunit GluN2B availability at synapses is reduced after TBI induction [14,15,20,21], evoking a disbalance in the synaptic/extrasynaptic signaling that triggers detrimental outputs to neurons, which in turn leads to neuronal malfunctioning [16,17,22,23].

The exocyst is a vesicle tethering protein complex composed of eight subunits: Sec3, Sec5, Sec6, Sec8, Sec10, Sec15, Exo70, and Exo84 [24,25,26], which oversees the initial contact of secretory vesicles with the plasma membrane during exocytosis [24,25,26,27]. Many cellular processes have been associated with the exocyst, including cell division, membrane growth, cell migration, cell–cell contact, signaling, and tissue morphogenesis, among others [28,29]. It is required in neuronal development, where it promotes dendritic morphogenesis [30,31] and axonal elongation [32]. Additionally, it is needed in glutamate receptor trafficking and stabilization in synapses [33,34].

Exo70 is one of the most studied exocyst subunits and is postulated to be a limiting factor in the functionality of the complex [35,36]. Exo70 is primarily localized at the plasma membrane [37,38] and facilitates the localization of the rest of the exocyst to specific sites where cargo exocytosis is required [39,40,41]. Exo70 has been shown to participate in neurite development and synapse formation/stabilization [32,37]. It is also known to interact directly with GluN1 and GluN2B during NMDAR trafficking and delivery into the synapse [34], which is supposedly in charge of basal and stimulated exocytosis [28].

The knowledge of the exocyst’s involvement in brain pathologies is minimal. The genetic anomaly of Sec15 and downregulation are involved in neurodevelopmental abnormalities related to language abilities [38,42,43]. SEC5 and SEC6 variants also affect brain development, presenting severe hypoplastic hippocampi and shrinking cerebellum [44,45]. Outside those reports, only one has studied the exocyst’s involvement in neurodegenerative diseases such as Alzheimer’s, where Sec6 has been associated with brain glucose metabolism abnormalities [46]. Given the role of the exocyst in trafficking glutamate receptors, and the known impact of oxidative stress on these receptors, further research into exocyst function in the context of oxidative-stress-related pathologies like TBI is warranted.

Traumatic brain injury induces neuronal damage that includes the redistribution of NMDA receptors away from synaptic sites, leading to detrimental neuronal consequences. Given the exocyst’s established role in constitutive trafficking and synaptic delivery of glutamate receptors, we previously investigated the involvement of the exocyst subunit Exo70 in mTBI and its potential subcellular redistribution. Our results demonstrate that, while total Exo70 expression remains unchanged following injury induction, subcellular fractionation reveals a significant redistribution of Exo70 from the microsomal fraction to the synaptic compartment after mTBI. Furthermore, within the synaptic compartment, we observed an increase in both the assembly of the exocyst complex and its interaction with the GluN2B subunit of the NMDA receptor [47].

In the present study, we tested this hypothesis using lentiviral tools to overexpress Exo70 on the hippocampus, which allowed us to assess spatial memory and NMDAR function in mice subjected to mTBI.

## 2. Materials and Methods

### 2.1. Antibodies

Primary antibodies: rabbit Exo70 1:1000 (Proteintech, Rosemont, IL, USA), goat GFP 1:1000 WB; 1:500 IHC (NovusBiologicals, Centennial, CO, USA), mouse HA 1:1000 (Cell Signalling, Danvers, MA, USA), mouse tubulin 1:1000 (NovusBio, Centennial, CO, USA), mouse β-actin 1:1000 (Sigma, Camas, WA, USA), rabbit GluN2B 1:1000 (Invitrogen, Waltham, MA, USA), rabbit GluN2B pY1472 1:1000 (Cell Signalling, Danvers, MA, USA), rabbit GluN2B pY1336 1:1000 (Invitrogen, Waltham, MA, USA), rabbit pCREB 1:1000 (NovusBiologicals, Centennial, CO, USA), rabbit pERK1/2 1:1000 (ThermoFisher Scientific, Waltham, MA, USA), mouse ERK1/2 1:1000 (ThermoFisher Scientific, Waltham, MA, USA). Secondary antibodies: anti-rabbit and anti-mouse HRP 1:5000 for WB (Jackson ImmunoResearch, Baltimore, PA, USA), anti-goat HRP 1:500 for IHC (Jackson ImmunoResearch, Baltimore, PA, USA) [47].

### 2.2. Animals

One-month-old C57BL/6J male mice were used in this study. Animals were housed up to 4 mice per cage with a 12:12 h light/dark cycle (light on at 8:00 a.m.) and provided food and water ad libitum. Animals were obtained from the Center for Innovation in Biomedical Experimental Models (CIBEM-UC) at the Pontificia Universidad Católica de Chile. Animals were handled in accordance with the National Institutes of Health guidelines (NIH Publications No. 8023, revised 1978, Baltimore, MD, USA). All the procedures were approved by the Pontificia Universidad Católica de Chile bioethics committee, registration number 180814017. The animals were deeply anesthetized and were euthanized by decapitation.

### 2.3. Lentiviral Transduction in HEK293 Cells

Third-generation lentiviral particles were packaged by and acquired from Applied Biological Materials (ABM, Canada) based on the lentiviral vector FUG-1D2A-Exo70-W [37]. The bicistronic lentiviral construct expresses a single transcript containing the GFP and the Exo70 ORF. The self-cleaving 1D/2A sequence between the GFP and Exo70 ORFs generates both proteins separately, with an HA-tagged Exo70 at the *N*-terminus. Control lentiviral particles carrying GFP were used as a control. Lentiviral expression was first evaluated in HEK293 cells by immunoblot. Cells were grown in DMEM supplemented with 10% fetal bovine serum, 100 U/mL ampicillin, and 100 μg/mL streptomycin (Gibco, New York, NY, USA) and maintained at 37 °C in an atmosphere of 95% air and 5% CO_2_. Cells were transduced with 3 µL of high-titer (1 × 10^9^ IU/mL) lentiviral suspension. Then, 72 h after transduction, cells were harvested for Western blot analysis. The blot analysis revealed that GFP is only expressed in the transduced cells, whereas no GFP signal was detected in the non-transduced cells (Figure S1).

### 2.4. Intrahippocampal Lentiviral Injection

Similar to HEK293 cells, mice were injected with either control lentivirus (LV-GFP) or overexpression lentivirus (LV-Exo70), but the latter was absent in non-injected animals. One microliter of high-titer (1 × 10^9^ IU/mL in serum-free DMEM with 5% PEG6000) lentiviral particles was injected bilaterally into the CA1 stratum radiatum region of the dorsal hippocampus of 4-week-old C57BL/6J mice using a stereotaxic device. Coordinates from bregma were 1.8 mm caudal, 1.5 mm lateral, and 1.5 mm ventral. The injection rate was 0.5 µL/min. The syringe was kept inside for 5 min to allow the proper diffusion of lentiviral particles [48,49]. After surgery, the mice were carefully observed during their recovery process on a thermal pad maintained at 37 °C. By immunohistochemistry, GFP+ cells were detected in both LV-GFP- and LV-Exo70-injected mice across a wide area of the CA1 region in the dorsal hippocampus. Lentiviral expressions were carried out for four weeks. The timeline of the experiments begins with intrahippocampal lentiviral injection in the two experimental groups (LV-GFAP and LV-Exo70). After viral expression for 4 weeks, these two groups were divided into two additional groups (Sham and mTBI). Thus, there are 4 experimental groups (Sham-GFP, mTBI-GFP, Sham-Exo70, and mTBI-Exo70), n = 5–6 for each group. The mTBI application protocol (see Section 2.7) lasted for 10 days. Seven days after finishing the mTBI protocol, behavioral analyses begin. The next day (and 2 days after) the animals are euthanized to perform electrophysiological experiments and Western blot analyses. This is similar to what was developed in [20].

### 2.5. Immunohistochemistry

Mice were anesthetized with ketamine (80 mg/kg) and xylazine (10 mg/kg) via intraperitoneal injection. Mice were then perfused intracardially with saline solution and PFA 4%. Brains were removed and postfixated overnight in PFA 4%. A sucrose gradient was performed consisting of 10%, 20% for two hours each at room temperature and 30% overnight at 4 °C. Then, 30 µm thick brain slices were cut in a cryostat and stored at −20 °C with OLMOS cryopreservation solution until use. Slices were permeabilized with 0.2% (*v*/*v*) Triton X-100 in PBS (PBS-T) for 30 min. Then, a 30 min incubation was carried out with H_2_O_2_ 3% to decrease the background. Slices were washed with PBS-T three times and blocked with 3% BSA for 1 h at room temperature. Slices were incubated overnight at 4 °C with GFP primary antibody diluted in PBS-T containing 3% BSA. After three PBS-T washes, slices were incubated with a secondary antibody in PBS-T containing 3% BSA for 2 h at room temperature [50]. The slices were then washed three times with PBS and distilled water. Chromogenic staining was developed using a DAB kit (ThermoFisher Scientific, Waltham, MA, USA). Finally, slices were mounted on gelatin-coated slides, and images were captured in an Olympus BX51 microscope equipped with a Micro-publisher 3.3 RTV camera (QImaging, Surrey, BC, Canada) using Q-imaging software (for Micro-publisher 3.3).

### 2.6. Western Blot Analysis

Dorsal hippocampal slices were prepared using a vibratome (BSK microslicer DTK-1500E, Ted Pella, Redding, CA, USA) and immediately processed. Tissues were homogenized in RIPA buffer (10 mM Tris-HCl, Triton X-100 0.5%, 1% NP-40, 5 mM EDTA, 1% sodium deoxycholate, and 1% SDS) supplemented with protease and phosphatase inhibitor mixture (Protease: Amresco, VWR Life Science; Phosphatase: 25 mM NaF, 100 mM Na_3_VO_4_, and 30 µM Na_4_P_2_O_7_) using a Potter homogenizer and then passed through a tuberculin syringe. Samples were centrifuged at 14,000 rpm at 4 °C for 10 min. Protein concentration was determined using a BCA protein assay kit (Pierce, ThermoFisher Scientific, Waltham, MA, USA). Samples were resolved by SDS-PAGE and transferred to PVDF membranes. Western blot was performed as previously described [47] with overnight incubation of primary antibodies at 4 °C and 2 h incubation of secondary antibodies at room temperature. Blots were developed using a chemiluminescence detection kit (Westar Sun, Cyanagen, Bologna, Italy). Images were obtained with a G:BOX Chemi XT4 Gel imaging system (Syngene, Bangalore, India).

### 2.7. Mild Traumatic Brain Injury (mTBI) Procedure

To induce mTBI, we used a modified Maryland’s weight drop model [51]. For this purpose, we adapted the impact device to fit the mice’s anatomy [15,47]. The impact energy was obtained from a 180 g steel ball accelerated by gravity along a 1 m rail disposed at a 60° angle from the horizontal. The rail curved at the bottom end to redirect the ball onto a chamber where it struck a coupling arm. The arm then accelerated to impact the malar processes bilaterally with two pistons. After 4 weeks of lentiviral expression, mice were subjected to mTBI. Animals were randomly assigned to receive either a sham or mTBI. First, mice were anesthetized with isoflurane using the open-drop method [52]. Animals were then gently restrained in the impact device using an elastic belt placed across the dorsal thorax, leaving the head free. In a frontal weight impact device, mice were subjected to 5 sessions of 3 blasts each with a 2-day interval. After each session, mice were monitored carefully until the anesthesia effect was finalized and returned to their home cage. Then, animals were kept in their home cage for 7 days before analysis. Sham animals were subjected to all procedures except injury induction.

### 2.8. Behavioral Tests

The Morris water maze (MWM) task was employed as a behavioral test of spatial memory. Mice were trained in a 1.1 m diameter circular pool (opaque water, 50 cm deep) filled with 19–21 °C water. A submerged 9 cm platform (1 cm below the surface, invisible to the animal) was used for training, with a maximum trial duration of 60 s and 10 s on the platform at the end of the trials. Mice were placed into the water facing the side walls, being transported there by hand from a holding cage. Each animal was trained to locate the platform. All procedures were conducted between 9:00 a.m. and 12:00 p.m. The test was performed with three trials per day for 5 consecutive days, and a probe test was conducted 24 h later on day 6, after the platform was removed. Swimming was monitored using an automatic tracking system (ANY-maze video tracking software (v 7.00), Stoelting Co, Wood Dale, IL, USA). This system measured the latency time (in seconds) required for the animal to reach the platform and the time spent in each quadrant (in seconds). After testing, the mouse was gently removed from the maze and returned to its cage.

Spatial acuity was calculated using the time mice spent in the quadrant after the platform was removed on the probe day, as well as the time mice spent in the platform area. Both times were multiplied to obtain the spatial acuity score. Data were plotted as the sum of escape latency (from the 5 days of learning phase) on the x-axis versus the spatial acuity score on the y-axis.

The week after MWM was performed, memory flexibility was tested using a modified MWM paradigm. Each animal was trained on one pseudo-random platform location daily for 4 days, with a new platform location assigned each day. Up to 15 training trials were performed daily until the criterion of 3 successive trials with an escape latency of <20 s was met (intertrial delay time = 20 min). Upon testing completion, mice were gently removed from the maze and returned to their cage. The animals were tested for the following location on the following day. Data were collected using a water maze video tracking system (ANY-maze video tracking software, Stoelting Co, Wood Dale, IL, USA).

### 2.9. Electrophysiology

Transverse slices (400 μm) from the dorsal hippocampus were cut under cold artificial cerebrospinal fluid (ACSF, in mM: 124 NaCl, 2.6 NaHCO_3_, 10 D-glucose, 2.69 KCl, 1.25 KH_2_PO_4_, 2.5 CaCl_2_, 1.3 MgSO_4,_ and 2.60 NaHPO_4_) using a Vibratome (BSK microslicer DTK-1500E, Ted Pella, Redding, CA, USA) and incubated in ACSF for 1 h at room temperature. In all experiments, 10 μM PTX was added to suppress inhibitory GABAA transmission. Slices were transferred to an experimental chamber (2 mL), superfused (3 mL/min, at room temperature) with gassed ACSF (using 95% O_2_/5% CO_2_), and visualized by trans-illumination with a binocular microscope (Amscope, Irvine, CA, USA). To evoke field excitatory postsynaptic potentials (fEPSPs), Schaffer collaterals were stimulated with bipolar concentric electrodes (Tungsten, 125 μm OD diameter, Microprobes) connected to an isolation unit (Isoflex, AMPI, Jerusalem, Israel). The stimulation was performed in the stratum radiatum within 100–200 μm from the recording site. Recordings were filtered at 2.0–3.0 kHz, sampled at 4.0 kHz using an A/D converter (National Instrument, Austin, TX, USA), and stored with the WinLTP program. The basal excitatory synaptic transmission was measured using an input/output curve protocol with a 10 s interval between stimuli. We used a high-frequency stimulation (HFS) protocol to generate LTP, consisting of 3 trains at 100 Hz stimuli with an inter-train interval of 10 s. In order to isolate NMDAR fEPSP, slices were incubated with 20 µM NBQX 30 min before testing started, and the antagonist was kept throughout the recording [20,22]. Data were collected and analyzed offline with pClamp 10 software (Molecular Devices, San Jose, CA, USA).

### 2.10. Image and Statistical Analysis

Densitometry analysis was carried out with ImageJ (NIH) software 1.54, and the data were normalized with specific loading control indicated in each figure legend. All data are expressed as mean ± SEM. For multiple-grouped experiments, statistical analysis was calculated using two-way ANOVA with Bonferroni correction for multiple comparisons. A *p* < 0.05 value was considered significant. GraphPad Prism 8 (v8.0.2) was used to carry out statistical analysis.

## 3. Results

We previously showed that Exo70 is redistributed into synapses after an mTBI insult [47]. This Exo70 redistribution triggers the exocyst complex assembly in the postsynaptic compartment, where the interaction between Exo70 and GluN2B increases upon mTBI induction. It has been reported that TBI induces the exit of GluN2B from the synapse [21], detrimental to neuron functionality [12]. Critically, mTBI is known to cause significant oxidative stress, characterized by an increase in reactive oxygen species (ROS) and a compromised antioxidant defense system [8]. This oxidative environment can directly impact NMDAR function, including GluN2B-containing receptors, potentially contributing to their altered trafficking and synaptic loss [9,11,18]. Our prior work has demonstrated that oxidative stress can change the distribution and function of synaptic proteins, including those involved in glutamate receptor trafficking [22]. Thus, Exo70 might play a role as a compensatory mechanism, stabilizing GluN2B at the postsynaptic density and preventing the development of TBI pathology, potentially by counteracting the downstream effects of oxidative stress on NMDAR trafficking. To test this hypothesis, we overexpressed Exo70 in CA1 neurons using lentiviral transduction. The lentivirus used in this work is a third-generation system based on human immunodeficiency virus, which grants a high biosafety level due to its lack of viral replication capacity (see Section 2). This system enables the long-term, stable expression of the protein of interest without significantly affecting neuronal network activity [53]. Mice injected with lentiviral systems have demonstrated stable lentiviral expression for months, showing even cognitive changes [49,54,55], and are a reliable tool for research on TBI physiology [56].

### 3.1. Evaluation of Exo70 Overexpression in Learning and Memory

**Figure 1:**
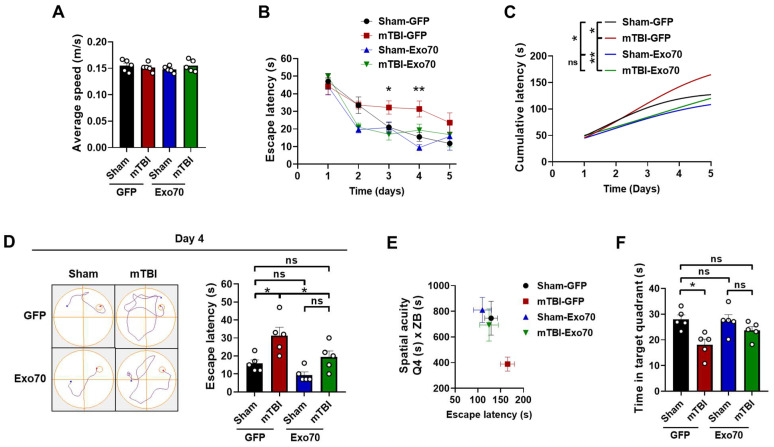
**Spatial learning and memory impairment are prevented by Exo70 overexpression in mTBI-induced mice.** Behavioral performance was tested using the MWM test. (**A**) Average swimming speed. (**B**) Escape latency (time to reach the hidden platform) of Sham and mTBI mice expressing GFP or Exo70. (**C**) Cumulative latency throughout the 5 days of the learning phase. (**D**) Representative swimming trajectories and escape latency for all experimental groups on day 4. (**E**) Spatial acuity for sham and mTBI mice with or without Exo70 overexpression. Spatial acuity was calculated using data from the learning phase and the probe trial (see Section 2), white circles indicate individual experiments. (**F**) Analysis of the time mice spent swimming in the area near the platform when it was removed on day 6. Values represent means  ±  SEM, n  =  5 mice per experimental group. Statistical differences were calculated by two-way ANOVA, followed by post hoc Bonferroni’s test. Curves were analyzed by Repeated Measures Two-way ANOVA. * *p* < 0.05, ** *p* < 0.01. ns: statistically non-significant difference.

Mice were bilaterally injected on postnatal day 30 with 1 µL of lentiviral suspension, and the expression was analyzed 30 days after using dorsal hippocampal samples from the injected mice. TBI diminishes spatial learning and memory [57,58]. The Morris water maze (MWM) assesses spatial memory hippocampal function. For this task, mice must learn the location of a hidden platform based on external cues. The swimming speed of the experimental groups was measured to detect possible motor problems. The groups evaluated do not show significant differences (main F: 0.20 LV, 0.16 mTBI) (Figure 1A). Sham-GFP mice showed a typical learning curve as expected for non-injured animals, and mTBI increased escape latency on day 3 and day 4 during the test. At the same time, Exo70 overexpression returned escape latency to normal (Figure 1B). Exo70 overexpression in Sham mice slightly but not significantly improved the escape latency on day 2 and day 4 of the test. As observed in Figure 1B, Sham-Exo70 mice showed a slight improvement in finding the hidden platform compared to Sham-GFP control animals. To evaluate if these differences could be significant, we fitted in a non-linear regression the escape latency from the learning phase and presented it in a cumulative manner. Cumulative latency showed that Exo70 overexpression reduced the escape time of both LV-Exo70 Sham/mTBI mice when compared to the control Sham-GFP mice group (Figure 1C), suggesting that Exo70 overexpression might enhance learning processes in the MWM test. Specifically, on day 4, the results show that Exo70 overexpression rescues the escape latency, as shown in Figure 1D (main F: 8.58 LV, 16.62 mTBI; interaction F: 0.79); representative swimming paths are also shown.

Next, we investigated the level of spatial acuity of these animals. Spatial acuity is a more sensitive parameter to measure spatial learning, representing the probability of finding the mice in a specific region around the hidden platform. Figure 1E shows the relationship between spatial acuity and the average escape latency of animals from the different experimental groups. The graph shows that mTBI-GFP mice are located in a region of the graph that corresponds to high escape latency values and low spatial acuity scores. In contrast, Sham-GFP and Sham-Exo70 mice show low escape latency values and high spatial acuity scores. Exo70 overexpression was able to rescue spatial acuity (Figure 1E). Twenty-four hours after the last trial of the learning phase, the platform was removed in a probe test that measured the time mice spent swimming in the former area near the initial platform location (a circular area with twice the platform radius). Sham-GFP and Sham-Exo70 mice spent more time in the platform area than TBI-GFP mice. At the same time, Exo70 overexpression rescued the memory deficit, demonstrating the inability of mTBI mice to remember the platform location and the improvement in TBI-Exo70 mice (main F: 1.67 LV, 12.38 mTBI; interaction F: 2.43) (Figure 1F).

**Figure 2:**
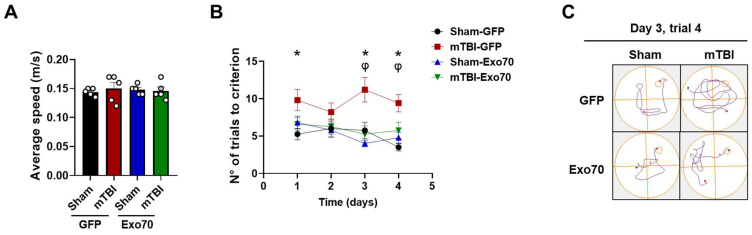
**Exo70 overexpression prevents memory flexibility impairment.** (**A**) Average swimming speed, white circles indicate individual experiments (**B**) Behavioral performance was tested using a modification of the MWM, memory flexibility tests (see Section 2). Memory flexibility was performed the week after the MWM test was carried out. The Y-axis corresponds to the mean number of trials that mice performed to reach the learning criterion (three consecutive < 20 s trials). (**C**) Representative swimming trajectories for trial 4 on day 3 of the test are shown. Values represent means  ±  SEM, n  =  5 mice per experimental group. Statistical differences were calculated by repeated measures Two-way ANOVA and post hoc Bonferroni’s test. Sham-GFP/TBI-GFP comparison: * *p* < 0.05. TBI-GFP/TBI-Exo70 comparison: φ *p* < 0.05.

Additionally, animals’ cognitive performance was evaluated using a modified spatial memory paradigm associated with episodic memory (memory flexibility), which is more sensitive in detecting hippocampal dysfunction [22]. Swimming speed was not altered between the four experimental groups (main F: 0.01 LV, 0.08 mTBI) (Figure 2A). The analysis of behavioral performance indicates that, almost every day, mTBI-GFP mice required more trials to achieve the learning criterion (see Section 2) than control Sham-GFP mice, while Exo70 overexpression prevented learning and memory impairments (Figure 2B). Figure 2C shows representative swimming paths.

### 3.2. Synaptic Transmission Evaluation

**Figure 3:**
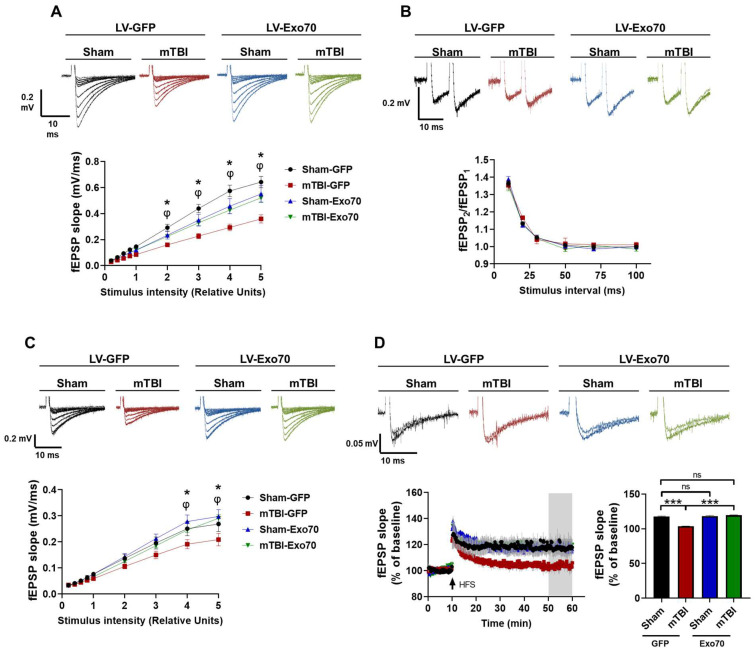
**Synaptic transmission damage is protected by Exo70 overexpression.** (**A**) Basal synaptic transmission in hippocampus from mTBI mice expressing GFP or Exo70. fEPSP slope induced by the input–output protocol to record total responses of CA3-CA1 synapses. Crescent stimuli recorded responses. (**B**) Paired-pulse facilitation (PPF) of fEPSP shows no alterations in presynaptic activity at CA3-CA1 synapses. (**C**) NMDAR-related synaptic transmission in hippocampus from mTBI mice expressing GFP or Exo70. Recordings were made by incubating slices with 20 µM NBQX. fEPSP slope induced by the input–output protocol to record NMDAR responses of CA3-CA1 synapses. Crescent stimuli recorded responses. (**D**) LTP was induced by high-frequency stimulation (HFS) in the hippocampal CA1 area, and the recording was conducted for 50 min. Quantification of fEPSP slope in the last 10 min of the recording. Traces of representative records are shown at the top of each figure. All graph values represent means  ±  SEM, n  =  6 slices per experimental group. Statistical differences were calculated by repeated measures ANOVA, followed by post hoc Bonferroni’s test. Sham-GFP/TBI-GFP comparison: * *p* < 0.05. TBI-GFP/TBI-Exo70 comparison: φ *p* < 0.05. The bar graph was analyzed using two-way ANOVA with Bonferroni’s correction. *** *p* < 0.001. ns: statistically non-significant difference.

One of the TBI cellular hallmarks is the alteration of synaptic transmission upon injury induction, both in basal and stimulated transmission. Therefore, we evaluated the total synaptic strength using input/output curves by stimulating Schaffer collaterals with crescent stimuli. A significant decrease in total response to different stimulus intensities was found in mTBI-GFP compared to Sham-GFP mice (Figure 3A), which were partially rescued by Exo70 intrahippocampal overexpression. Complementarily, we evaluated presynaptic function using the paired-pulse facilitation assay, and no differences were found between any of the experimental groups (Figure 3B).

The NMDAR-dependent response in basal synaptic transmission was also assessed by incubating slices in Mg^2+^-free ACSF with 20 µM NBQX, an AMPA receptor antagonist, and recording input/output curves. Similar to total response recording, mTBI decreased NMDAR-dependent synaptic responses, while overexpression of Exo70 rescued synaptic strength in response to all stimuli applied (Figure 3C). Next, synaptic plasticity was evaluated by examining the magnitude of LTP in hippocampal CA3-CA1 transmission, which also correlates with learning and memory [59]. Using a high-frequency stimulation protocol (3 trains at 100 Hz), LTP induction was compromised in TBI-GFP compared to Sham-GFP control mice (Figure 3D). TBI-GFP mice exhibited potentiation but were unable to maintain LTP, whereas Sham-Exo70 mice could induce LTP at a similar rate to control animals. Finally, TBI-Exo70 mice were able to induce and maintain LTP throughout the protocol (main F: 466.97, LV, 35.69 mTBI; interaction F: 178.67) (Figure 3D). These results reinforce the rescued memory of the mTBI animals injected with LV-Exo70.

### 3.3. NMDAR Synaptic Availability

**Figure 4:**
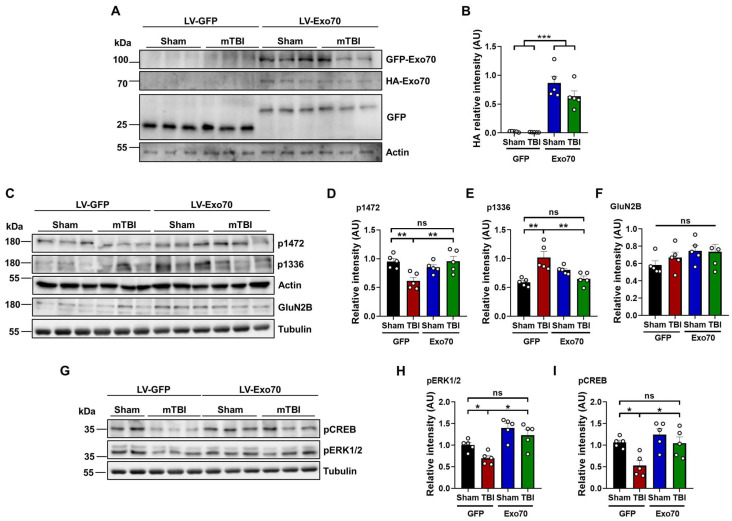
**Exo70 overexpression reinforces NMDAR synaptic availability upon mTBI induction.** Samples from the dorsal hippocampus were analyzed by Western blot. Here, 30 µg of protein samples were resolved in 10% SDS-PAGE and transferred to PVDF membranes. Tubulin and Actin were used as loading controls. (**A**) Characterization of the lentiviral expression in all experimental groups using GFP and HA antibodies. (**B**) Densitometric analysis of HA-Exo70. (**C**) Western blot analyzing NMDAR synaptic localization after mTBI in injected mice. Samples were analyzed using GluN2B p1472, GluN2B p1336, and total GluN2B antibodies. (**D**–**F**) Densitometric analysis normalized with loading controls before normalization with Sham-GFP control samples. (**G**) Western blot analyzing NMDAR synaptic-signaling-related proteins after mTBI induction in Exo70-overexpressing samples. Samples were analyzed using pCREB and pERK1/2 antibodies. (**H**,**I**) The graphs show the densitometric analysis normalized with loading controls before normalization with Sham-GFP control samples. White circles indicate individual experiments. Values represent means  ±  SEM, n  = 5 mice per experimental group. Statistical differences were calculated by two-way ANOVA, followed by post hoc Bonferroni’s test. * *p*  <  0.05, ** *p*  <  0.01, *** *p* < 0.001, ns: statistically non-significant difference.

One of the key features of NMDARs is that they are found in both synaptic and extrasynaptic membranes. Several reports demonstrate that tyrosine phosphorylation of the NMDAR GluN2B subunit is strongly associated with the surface expression of this receptor. For instance, the phosphorylation of tyrosine 1472 determines the surface expression of NMDARs in the synaptic zone [60], and phosphorylated tyrosine 1336 is associated with the enrichment of the receptor in extrasynaptic membranes [61]. Thus, we asked whether the previously observed increased interaction between Exo70 and GluN2B [52] could be part of a compensatory mechanism by which GluN2B is intended to be stabilized in the synapse, as learning and memory, as well as NMDAR-associated synaptic transmission, were rescued by Exo70 overexpression. First, samples from the dorsal hippocampus were analyzed by immunoblot to evaluate the proper expression of the lentiviral construct. Soluble GFP was present in mice from all experimental groups, but only GFP-Exo70 was found in both Sham and mTBI mice injected with LV-Exo70 (Figure 4A); again, both expected GFP bands (see Section 2) were observed in all hippocampal samples. Accordingly, only HA-Exo70 was observed in LV-Exo70 samples, indicating Exo70 overexpression (main F: 94.84 LV, 2.60 mTBI; interaction F: 2.10) (Figure 4B).

Using GluN2B p1472 and p1336 antibodies, it was detected that mTBI reduced the p1472 signal compared to control, whereas TBI-Exo70 samples showed a restored signal. Exo70 overexpression in Sham mice did not alter the phosphorylation state of Y1472 (main F: 3.52 LV, 3.16 mTBI; interaction F: 12.30) (Figure 4C,D). Contrary to p1472, mTBI increased the phosphorylation of Y1336, which was partially decreased by Exo70 overexpression. Sham-Exo70 mice showed an apparent increase in the p1336 signal but without statistical significance (main F: 1.68 LV, 5.31 mTBI; interaction F: 23.20) (Figure 4C,E). GluN2B total protein levels did not change in the experimental groups (main F: 2.96 LV, 0.30 mTBI; interaction F: 0.53) (Figure 4C,F). These results suggest that a disbalance of synaptic/extrasynaptic GluN2B occurs with mTBI [20] but also indicate that Exo70 overexpression can restore the balance of the synaptic distribution of this NMDAR subunit.

Because an NMDAR synaptic/extrasynaptic disbalance was found, we asked whether these results could correlate with the downstream synaptic signaling through ERK1/2 and CREB. Thus, samples were analyzed using specific antibodies showing phosphorylation of residues related to NMDAR synaptic activity (Figure 4G). The PERK1/2 signal was reduced in mTBI-GFP mice compared to control animals, while mTBI-Exo70 mice showed a restored signal (main F: 25.36, LV: 6.54 mTBI; interaction F: 0.57) (Figure 4H). Similar results were observed with the pCREB antibody, where Exo70 overexpression rescued the phosphorylation state of this protein (main F: 8.67 LV, 9.53 mTBI; interaction F: 2.04) (Figure 4I). Finally, neither pERK1/2 nor pCREB signal was altered with Exo70 overexpression in Sham animals. These results support the idea that mTBI evokes an exit of GluN2B from the synapse and that the synaptic/extrasynaptic imbalance is restored or prevented when Exo70 is overexpressed.

## 4. Discussion

The exocyst complex plays a crucial role in tethering secretory vesicles during polarized exocytosis, contributing to specialized membrane processes in neurons, including the trafficking and synaptic availability of ionotropic glutamate receptors [28,33,34,62]. This study reveals a novel function for the exocyst subunit Exo70 in mitigating the detrimental effects of mild traumatic brain injury (mTBI), a condition increasingly recognized for its complex cellular consequences, including significant oxidative stress.

We previously reported that Exo70 redistributes to hippocampal synapses in mice subjected to mTBI, with a concomitant increase in its interaction with the GluN2B subunit of NMDARs [47]. We hypothesized that this redistribution might represent a compensatory mechanism to stabilize NMDARs at the synapse, thereby counteracting the synaptic loss of these receptors, which is known to occur following TBI [21]. To test this, we overexpressed Exo70 in CA1 neurons of the dorsal hippocampus, a region critical for spatial memory [63], using a lentiviral approach that ensures stable, long-term expression without significantly disrupting neuronal network activity [53].

Our findings strongly support this hypothesis. Exo70 overexpression prevented mTBI-induced cognitive impairments in spatial learning and memory, as assessed by the Morris water maze (MWM) and its more sensitive variant, which evaluates memory flexibility. This cognitive protection correlated with the preservation of both basal and NMDAR-dependent hippocampal synaptic transmission, which are typically compromised following mTBI [64,65], as shown in Figure 3. Furthermore, Exo70 overexpression prevented the mTBI-induced impairment in long-term potentiation (LTP), a cellular correlate of learning and memory [59,66,67].

A key aspect of mTBI pathophysiology is the disruption of NMDAR subunit localization. We observed a decrease in the synaptic form of NMDARs, as indicated by reduced phosphorylation of GluN2B at tyrosine 1472, and an increase in the extrasynaptic form, characterized by increased phosphorylation at tyrosine 1336, in mTBI animals, consistent with previous reports [20,21]. This shift towards extrasynaptic NMDARs can trigger detrimental signaling cascades, impacting neuronal survival [12,22,23]. Notably, this shift in NMDAR localization is likely exacerbated by the oxidative stress inherent to mTBI [8] (from the Introduction). Oxidative stress can directly modify NMDAR function and trafficking, potentially promoting the internalization of synaptic receptors [9,10].

Exo70 overexpression in mTBI animals reversed this imbalance, restoring the phosphorylation levels of GluN2B at both Y1472 and Y1336 to near-control levels. This suggests that Exo70 promotes the stabilization of GluN2B-containing NMDARs at the synapse, potentially counteracting the effects of oxidative stress on receptor trafficking. While Exo70 overexpression in sham animals showed a slight, non-significant increase in pY1336, suggesting a potential role in basal extrasynaptic NMDAR trafficking as well [28,33], the most striking effect was the normalization of this balance in the mTBI context.

This restoration of NMDAR localization likely contributes to the observed preservation of downstream signaling. mTBI reduced the phosphorylation of ERK1/2 and CREB, key signaling molecules downstream of synaptic NMDAR activation, consistent with previous findings in chronic TBI models [67,68,69,70] and as shown in Figure 4I. Exo70 overexpression restored the phosphorylation of these proteins, further supporting the idea that it promotes functional synaptic NMDAR signaling.

Although our paired-pulse facilitation data suggest that the primary locus of Exo70’s protective effect in our model is postsynaptic, we cannot rule out a presynaptic contribution, as Exo70 is present in both compartments [37]. Future studies could specifically address the pre- versus postsynaptic contributions of Exo70 using more targeted approaches.

The connection between Exo70, NMDAR trafficking, and dendritic spine dynamics warrants further investigation. mTBI is associated with dendritic spine loss and degeneration [71,72], which correlates with impaired synaptic transmission [73]. Exo70 is known to be involved in spine formation and stabilization [37], and the exocyst complex is recruited to the postsynapse upon stimulation, promoting synapse formation [68]. While our data do not show an enhancement of basal synaptic transmission with Exo70 overexpression in sham animals, suggesting that an “insult” like mTBI might be necessary to engage Exo70’s protective mechanisms fully, it is plausible that Exo70’s effects on NMDAR trafficking contribute to the maintenance of spine integrity following mTBI. Future studies employing techniques like two-photon microscopy could directly examine the impact of Exo70 overexpression on spine density and morphology in the context of mTBI and oxidative stress.

The precise mechanism by which Exo70 exerts its protective effects in mTBI remains to be fully elucidated. However, there is an established role of oxidative stress in mTBI pathophysiology [8] and known sensitivity of NMDARs to redox modulation [10,11].

Several potential mechanisms could explain Exo70’s protective effects in the context of oxidative stress. Firstly, Exo70, or other exocyst components, may directly interact with oxidized proteins involved in NMDAR trafficking, thereby preventing dysfunction. While Exo70 itself is not a known antioxidant, it could potentially interact with proteins that are affected by oxidation. Secondly, Exo70 may influence the activity of other trafficking proteins, such as adaptors or motors, which are themselves sensitive to oxidative stress, thereby indirectly protecting NMDARs. Lastly, although perhaps less likely given Exo70’s known function, it is formally possible that Exo70 overexpression could indirectly enhance local antioxidant defenses at the synapse; however, this would require further investigation, potentially utilizing experiments with genetically encoded redox sensors targeted to the synapse to assess the local redox environment in the presence and absence of Exo70 overexpression [69,70].

One of the most relevant limitations of our study is related to the exploration of the effects of Exo70 deficiency on neuronal cells, and this may be relevant when defining that a decrease in Exo70 levels could enhance the negative effects of mTBI. Another limitation of our study is the lack of definition of the cell type on which Exo70 acts. Our proposal is that the effects are mainly at the neuronal level, but the possibility is open that part of what is observed is mediated, at least in part, by glial cells, including astrocytes.

Future studies should also investigate the potential therapeutic implications of targeting Exo70 in mTBI. Could pharmacological modulation of Exo70 activity or gene therapy approaches similar to the one used here provide a novel avenue for protecting against the cognitive and synaptic deficits associated with mTBI? Furthermore, investigating the role of other exocyst subunits in the context of mTBI and oxidative stress could reveal additional therapeutic targets.

In conclusion, our findings demonstrate a novel neuroprotective role for Exo70 in mTBI, likely mediated by its ability to stabilize synaptic NMDARs and preserve downstream signaling in the face of oxidative stress. This work highlights the exocyst complex as a potentially critical regulator of neuronal function in both health and disease. It opens up new avenues for research into the treatment of mTBI and other neurological disorders characterized by oxidative stress and NMDAR dysfunction.

## 5. Conclusions

**Figure 5:**
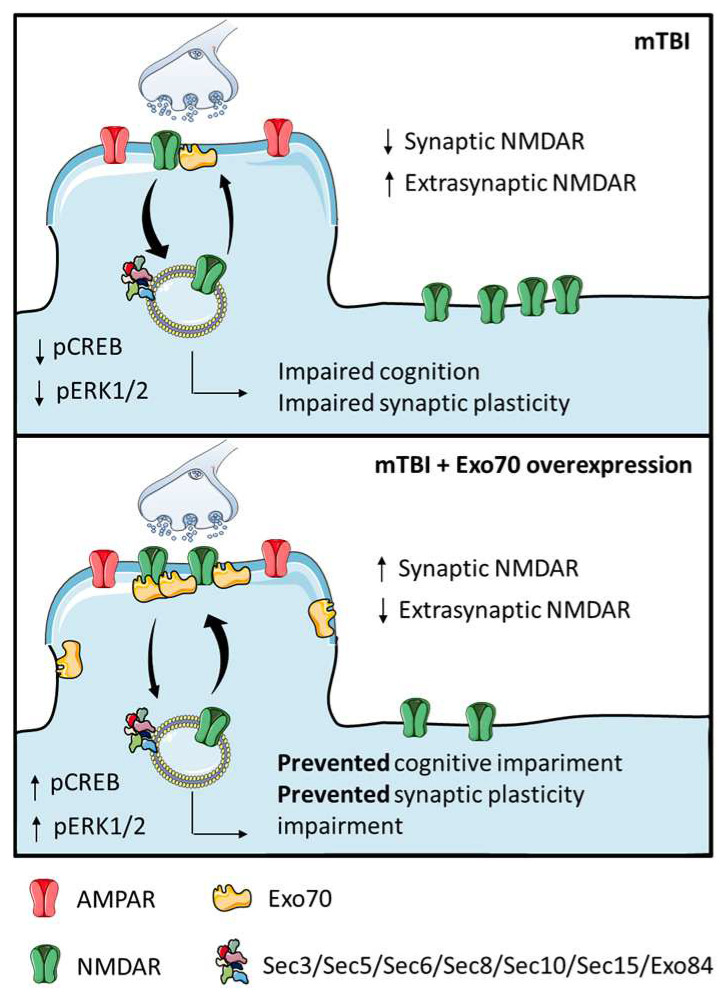
Schematic summary of Exo70 overexpression effects in our mTBI model.

The summary in Figure 5 illustrates that our repeated mTBI protocol, a condition recognized for inducing significant oxidative stress that can overwhelm cellular antioxidant capacity, promotes the exit of GluN2B from synapses.

Repeated mTBI, eliciting substantial oxidative stress, induces GluN2B translocation from synapses, likely exacerbated by the pro-oxidative environment impacting NMDARs/trafficking. This aberrant localization attenuates synaptic signaling, reducing basal glutamatergic transmission, LTP, and NMDAR-dependent functions vulnerable to redox dysregulation in TBI. This synaptic dysfunction correlates with spatial memory deficits in mTBI-GFP mice. Exo70 overexpression counteracts these effects, preserving synaptic NMDAR localization and signaling, potentially by stabilizing these components against oxidative stress. This restored signaling mitigates redox imbalance sequelae, promoting near-normal neuronal activity and survival in the CA3-CA1 network, evidenced by preserved LTP and recovered cognition despite mTBI and its oxidative burden.

## Data Availability

The datasets generated during and/or analyzed during the current study are not publicly available due to constant changes in the computational services but are available from the corresponding author on reasonable request.

## Supplementary Materials

The following supporting information can be downloaded at: https://www.mdpi.com/article/10.3390/antiox14060640/s1. Figure S1: Characterization of the lentiviral system expressing Exo70.
