# Supplementary material for: Exo70 Protects Against Memory and Synaptic Impairments Following Mild Traumatic Brain Injury
Source: Antioxidants (Basel). 2025 May 26;14(6):640. doi: 10.3390/antiox14060640 (PMC12189859; doi:10.3390/antiox14060640)
Supplement: Supplementary file 1 [file antioxidants-14-00640-s001.zip › antioxidants-3554022-supplementary.pdf]

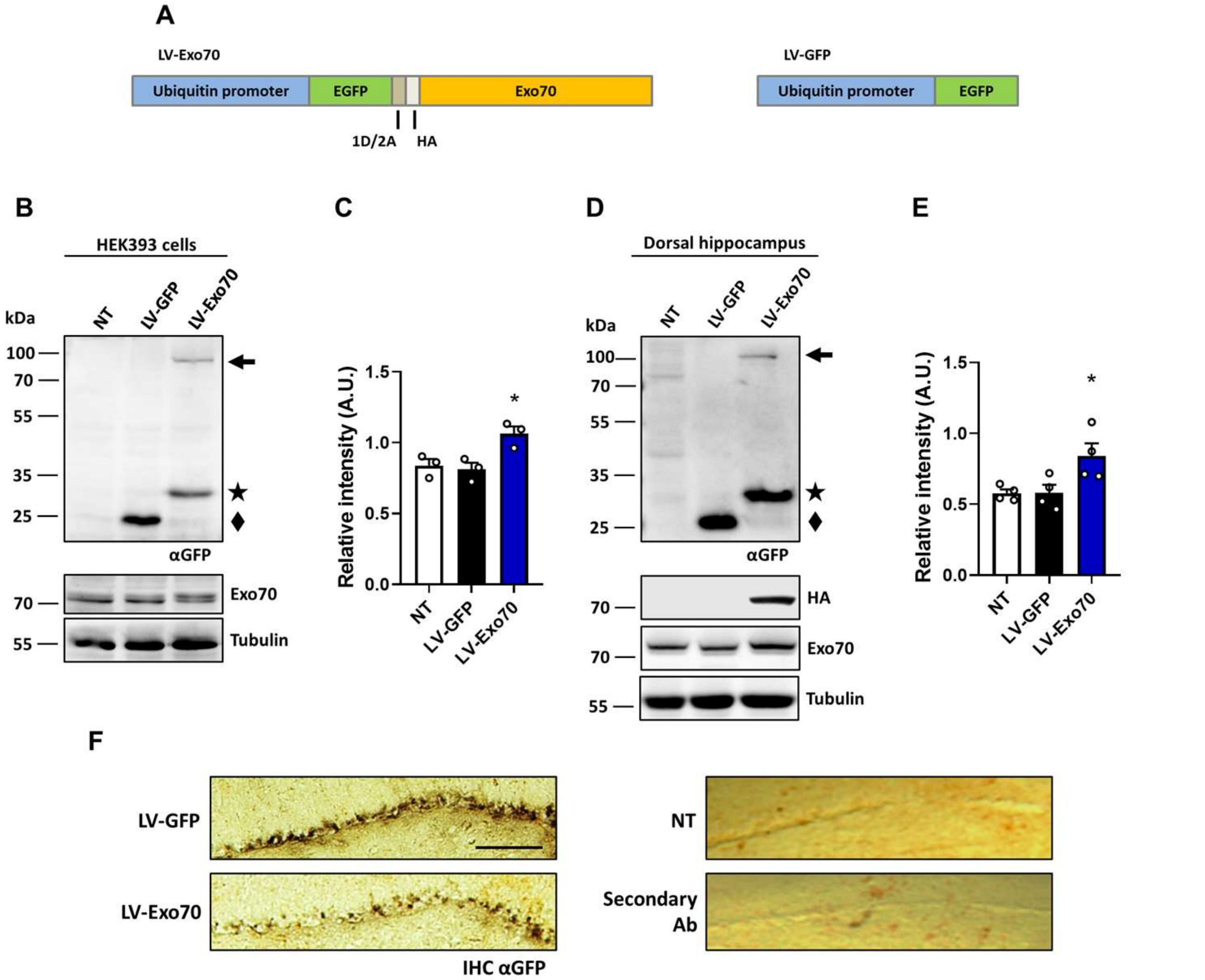

Figure Supplementary S1: Characterization of the lentiviral system expressing Exo70. (A) Scheme depicting both lentiviral constructs used in this study. (B) Immunoblot analysis of HEK293 cells transduced with 3 µl of lentiviral suspension. Expression was carried out for 72h. 30 µg of protein samples were resolved in 10%SDS-PAGE and transferred to PVDF membranes. Membranes were stripped and tested again with the indicated antibodies. (C) One-month-old mice were injected intrahippocampally with 1 µL of LV-GFP, LV-Exo70 lentiviral suspension, or PBS (NT: non-transduced). Expression was carried out for 30 days. Immunoblot analysis of dorsal hippocampal samples from injected mice. 30 µg of protein samples were resolved in 10% SDS-PAGE and transferred to PVDF membranes. Membranes were stripped and tested again with the indicated antibodies. (C-E) Densitometric analysis of Exo70. Signal intensity was normalized with Tubulin band intensity. The analyses show Exo70 overexpression only in LV-Exo70 transduced samples. Values represent means ± SEM. n = 3 independent experiments with HEK293 cells, n = 4 mice per experimental group. Statistical differences were determined by an unpaired t-test comparing LV-GFP and LV-Exo70 samples. $^*p < 0.05$. (F) Classical IHC was carried out using a GFP antibody. Representative images of CA1 hippocampal region showing GFP signal. Scale bar: 100 µm.
